# Real-world comparison of Docetaxel versus new hormonal agents in combination with androgen-deprivation therapy in metastatic hormone-sensitive prostate cancer descrying PSA Nadir ≤ 0.05 ng/ml as marker for treatment response

**DOI:** 10.1007/s00345-022-04189-8

**Published:** 2022-10-26

**Authors:** Mona Kafka, Thomas Burtscher, Josef Fritz, Maximilian Schmitz, Jasmin Bektic, Michael Ladurner, Wolfgang Horninger, Isabel Heidegger

**Affiliations:** 1 Department of Urology, Medical University Innsbruck, Innsbruck, Austria; 2 Department of Medical Statistics, Informatics and Health Economics, Medical University Innsbruck, Innsbruck, Austria

**Keywords:** Metastatic hormone sensitive prostate cancer, Docetaxel, NHT, Clinical outcome, Biomarker

## Abstract

**Propose:**

Using Docetaxel chemotherapy or new hormonal agents (NHT) to intensify upfront systemic therapy resulted in improved survival rates compared to androgen deprivation monotherapy (ADT). Hence, combination therapies have become the new standard of care (SOC) in metastatic hormone-sensitive prostate cancer (mHSPC). However, head-to-head trails comparing different therapies as well as treatment-guiding biomarkers are still lacking. Thus, the aim of the present study was to compare clinical outcomes of Docetaxel versus NHT therapy in the real-world setting as well as to elaborate biomarkers predicting clinical outcome.

**Methods:**

We retrospectively assessed overall-survival (OS), progression-free survival 1 and 2 (PFS1/2) and time to progression (TTP) in 42 patients treated by either ADT + NHT or ADT + Docetaxel. In addition, we investigated clinical prognostic biomarkers.

**Results:**

Our survival analysis revealed 3-year OS of 89.4% in the NHT group compared to 82.4% in the Docetaxel group. 3-year PFS1 was 59.6% in the NHT group compared to 32.2% in the Docetaxel group and the TTP was 53.8% vs 32.2% (pOS = 0.189; pPFS1 = 0.082; pTTP* = *0.055). In addition, castration-resistance occurred more often in the Docetaxel group (78.6% vs 25%, *p = *0.004). Interestingly, a PSA-Nadir ≤ 0.05 ng/ml during therapy was associated with increased survival rates (*p < *0.001) while PSA levels at primary diagnosis had no influence on therapy outcome. Furthermore, a thyroid-stimulating hormone (TSH) increase during therapy was associated with improved clinical outcome (*p = *0.06).

**Conclusion:**

We observed a trend towards a higher benefit of NHT as first-line treatment compared to Docetaxel in men with mHSPC. Of note, a PSA-Nadir ≤ 0.05 ng/ml or a TSH-increase during therapy were predictors for therapy response.

## Introduction

Prostate cancer (PCa) is the second most common cancer in men with about 1.4 million new cases and more than 375.000 deaths in 2020 [1]. While patients with localized disease usually have good prognosis, cancer-specific survival (CSS) declines dramatically in metastatic disease. For decades, androgen deprivation therapy (ADT) was the standard of care (SOC) for patients with metastatic PCa [2]. Based on the convincing data of recent studies, treatment landscape of metastatic hormone-sensitive PCa (mHSPC) has changed. First, Sweeney et al. showed in the CHAARTED trial that the combination of ADT + Docetaxel leads to a significant reduction of the risk of death by 28% [3]. Further, the LATITUDE trial yielded similar results for the combination of ADT and Abiraterone acetate/prednisolone (AAP) in patients with newly diagnosed high-risk mHSPC (34% risk reduction of death) [4]. Finally, in 2019 two trials demonstrated a 33% reduction of risk of death in patients treated with a combination of ADT plus Enzalutamide (Enza) or Apalutamide (Apa) [5, 6]. Based on these results, management in mHSPC has been significantly modified the past years leading to the fact that either upfront NHT or chemotherapy in addition to ADT represents the SOC. Based on comparable response rates in registration trials and adverse events several therapeutic options can be offered claiming for biomarkers to pursue a personalized treatment approach. Although indirect comparison analyses have been used and validated to compare outcomes from randomized-controlled trials, this approach falls short of a direct (head-to-head) treatment comparison.

Thus, the present study aimed to (1) perform a comparison of ADT + NHT vs. ADT + Docetaxel in patients from a European real-world high volume center as well as to (2) assess biomarkers predicting therapy response to combinational therapy.

## Patients and methods

### Patient cohort

After obtaining a positive vote of the local ethical committee (vote number: 1395/2020) a retrospective single-center study was performed at the Medical University Innsbruck, Austria. A total of 42 men diagnosed with mHSPC between 2014 and 2020 were included. All patients were eligible to receive a first-line therapy consisting of ADT combined with either Docetaxel (6 cycles á 75 mg/m^2^ KOF 3-weekly) or a novel hormonal therapy (NHT) (AAP or Apa). Patient data were extracted into an excel spreadsheet and follow-up was conducted until a cut-off date (December 31, 2021). Clinical outcome was analyzed by assessing overall survival (OS), progression-free survival 1/2 (PFS1/2) and time to progression (TTP).

### Statistical analysis

Baseline patient characteristics were tabulated separately for patients treated with NHTs vs. Docetaxel, using absolute and relative frequencies for categorical variables. For continuous variables, the median as well as minimum to maximum (Min–Max) parameters were used. Clinical outcomes OS, PFS1/2 and TTP were analyzed using Kaplan–Meier survival analysis, and the influence of various factors on survival was tested using log-rank tests. Univariate and multivariable-adjusted hazard ratios (HRs) were obtained from Cox proportional hazards models. Multivariable adjustment was done for age and PSA levels at therapy start, the only variable for which we detected a statistically significant difference between the NHT and Docetaxel groups. PFS was assessed from the starting date of combination therapy while TTP refers to the beginning of ADT. Due to the highly skewed distribution of most of the analyzed continuous biomarkers, those variables were dichotomized for the survival analysis using clinically meaningful cut-off values. All statistical tests were two-sided at a significance level of 0.05. SPSS, version 26.0 (IBM Corp., Armonk, NY, USA) was used for statistical analysis.

## Results

### Baseline patient characteristics

All patients had an initial histopathological diagnosis of adenocarcinoma of the prostate and presented with mHSPC. Two cohorts were formed based on the systemic therapy added to ADT (NHT + ADT vs Docetaxel + ADT). Cohort 1 consisted of 24 patients (10 AAP, 14 Apa), while cohort 2 included 18 patients treated with 6 cycles Docetaxel in addition to ADT. Baseline patient characteristics were well balanced between groups (Table1). The only variable for which groups differed significantly were PSA levels at therapy start (1.6 ng/ml vs. 6.07 ng/ml, *p = *0.01). Median age at diagnosis was 69.7 years with a median PSA of 29.3 ng/ml at primary diagnosis. The majority of patients presented with high-risk, locally advanced PCa comprising 71.4% with ISUP 4 or 5 in primary biopsy. Of note, 75% of the patients had a positive digital rectal examination (DRE) status. Most patients had a good performance status reflected by a Charlson Comorbidity Index (CCI) of 8.6 not differing among groups, indicating that CCI did not influence therapy selection in our collective. Of importance, we modified the CCI by 6 points remitting due to malignancy, as it was a constant variable in all patients. 42.1% of patients underwent a previous local therapy (radical prostatectomy (RPE) or external beam radiation therapy (EBRT)), while 57.9% presented with de-novo metastatic disease. In addition, patients were classified according to the volume status defined by CHAARTED as well as the LATITUDE risk criteria as previously described emphasizing that high risk/high volume patients were well balanced among the treatment groups [3, 4]. Concerning metastatic load, we observed that the Docetaxel group had a higher, but not statistically significant, metastatic status (bone: 66.7% vs 83.3%, lymph nodes: 62.2% vs 83.3%, visceral: 20.8% vs 22.2%). Furthermore, the Docetaxel group included more de-novo metastasized patients compared to the NHT group (72.2% vs 45.8%, *p* value: 0.120).

**Table 1:** Baseline patient characteristics

Variable	Total cohort (*N = *42)			NHT (*N = *24)			Docetaxel (*N = *18)			Comparison between groups
	Median	Min–Max	*N*	Median	Min–Max	*N*	Median	Min–Max	*N*	*p* value^a^
Age at diagnosis (years)	69.7	47–82		65.8	47–82		71.1	58.5–77.2		0.446
PSA at diagnosis (ng/ml)	29.3	1.9–1153		31.3	1.9–1153		23.9	4.1–600		1.000
PSA at therapy start (ng/ml)	3.6	0.01–57.6		1.6	0.01–17.7		6.07	0.49–57.6		0.010
Time from ADT to systemic therapy (weeks)	8.07	2–23		9.48	4–23		6.28	1–14		0.096
Modified Charlson Comorbidity Index	8.6	6–12		8	6–12		9	7–11		0.765
ISUP1	4.8%		2	8.3%		2	0%		0	0.631
ISUP2	16.7%		7	20.8%		5	11.1%		2	
ISUP3	7.1%		3	4.2%		1	11.1%		2	
ISUP4	35.7%		15	37.5%		9	33.3%		6	
ISUP5	35.7%		15	29.2%		7	44.4%		8	
Positive biopsy cores	68.7%	20–100%		81.7%	46–100%		66.7%	20–100%		0.279
Previous local therapy	42.9%		18	54.2%		13	27.8%		5	0.196
RPE:	30.9%		13	41.7%		10	16.7%		3	
EBRT:	11.9%		5	12.5%		3	11.1%		2	
De-novo metastasized	57.1%		24	45.8%		11	72.2%		13	0.120
Suspicious DRE	75%		21	75%		12	75%		9	1.000
Disease volume (chaarted)/ risk (latitude*)*										
Low risk/low volume	42.5%		17	45.5%		10	38.9%		7	0.570
Low risk/high volume	5%		2	9.1%		2	0%		0	
High risk/low volume	7.5%		3	9.1%		2	5.6%		1	
High risk/high volume	45%		18	36.4%		8	55.6%		10	
Lymph node metastases (cN Stage)	71.4%		30	62.5%		15	83.3%		15	0.180
Bone metastases	73.8%		31	66.7%		16	83.3%		15	0.299
Visceral metastases	21.4%		9	20.8%		5	22.2%		4	1.000

DRE: Digital rectal examination, ISUP: International Society of Uropathology, PSA: Prostate-specific antigen

^a^*p* values were calculated using Fisher’s exact test for categorical, and Mann–Whitney *U* test for continuous variables

### Survival analyses

**Fig. 1:**
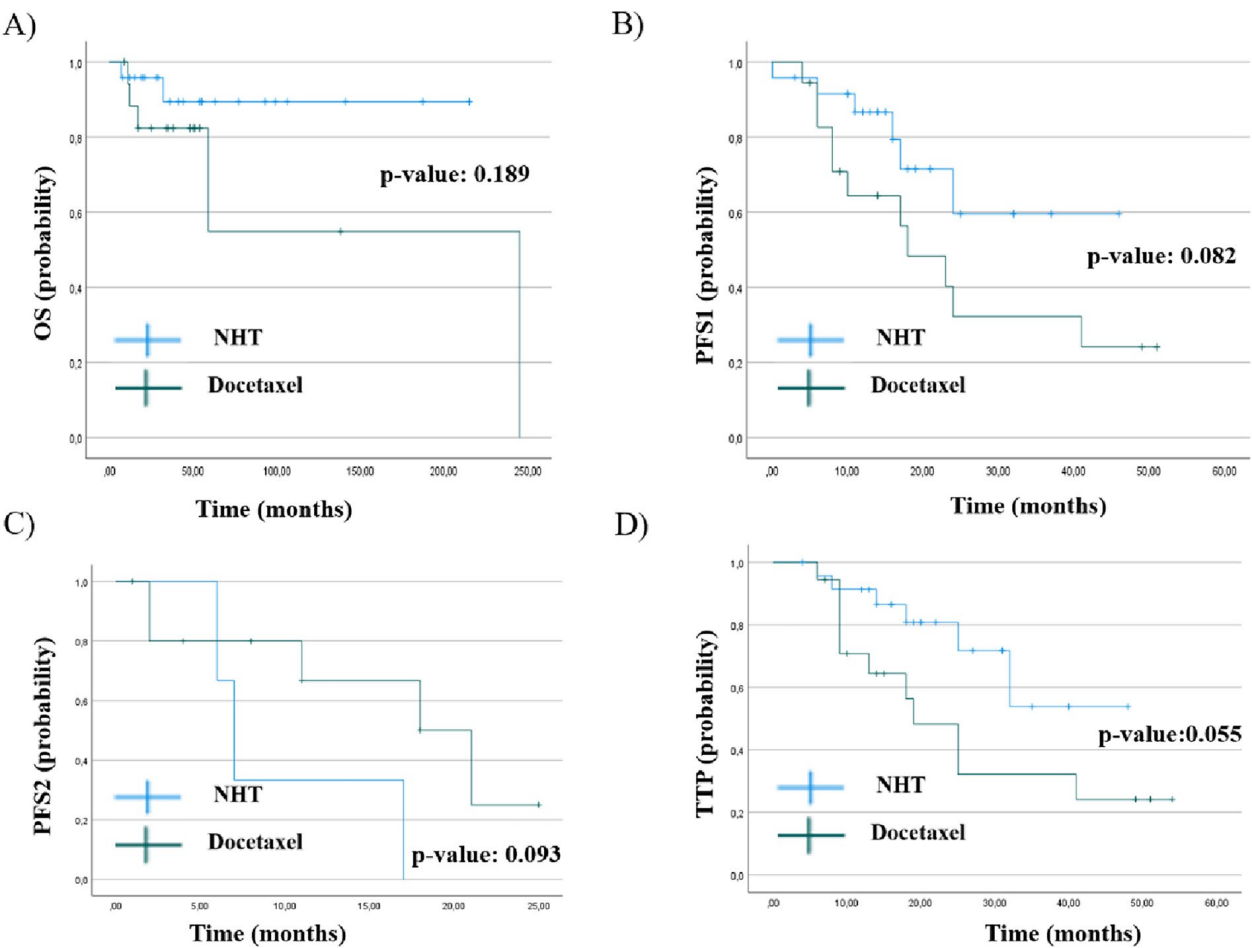
Kaplan-Maier curves of OS (**A**), PFS1 (**B**), PFS 2 (**C**) and TTP (**D**).The blue line depicts the NHT group and the green one the Docetaxel group. Ticks represent censored cases. *p* value from log-rank tests are as followed: OS: *p* value: 0.189, HR = 2.99, 95% CI 0.54–16.57, PFS1: *p* value: 0.082, HR = 2.35, 95% CI 0.86–6.42, PFS2: *p* value: 0.093 HR = 0.278, 95% CI 0.055–1.399 and TTP: *p* value: 0.055, HR = 2.53, 95% CI 0.92–6.94

Therapy outcomes of patients stratified according to systemic therapy are displayed in Fig. 1. Interestingly, we observed a trend towards better clinical outcomes in terms of OS, PFS 1 and 2, and TTP in the NHT group however without reaching statistical significance possibly caused by the limited statistical power due to limited sample size (OS: *p*_log-rank test_ = 0.189, Hazard ratio (HR) = 2.99, 95% CI 0.54–16.57; PFS1: *p*_log-rank test_ = 0.082, HR = 2.35, 95% CI 0.86–6.42; PFS2: *p* value: 0.093, HR = 0.28, 95% CI 0.06–1.40; TTP: *p*_log-rank test_ = 0.055, HR = 2.53, 95% CI 0.92–6.94). Results did not substantially change when adjusting models for age and PSA levels at treatment start (OS: HR = 4.96, 95% CI 0.67–36.52, *p = *0.116; PFS1: HR = 2.48, 95% CI 0.88–6.97, *p = *0.085; PFS2: HR = 0.67, 95% CI 0.07–6.14, *p = *0.720; TTP: 2.64, 95% CI 0.94–7.38, *p = *0.065).

3-year OS was 89.4% in the NHT group vs. 82.4% in the Docetaxel group. The 3-year PFS1 was 59.6% in the NHT group vs. 32.2% in the Docetaxel group, and for TTP it was 53.8% vs. 32.2%, respectively. Numbers available for analysis of PFS2 (only those patients with a lack of progress in the first-line therapy) were too small for a detailed analysis. Castration-resistance was reached statistically significant more often in the Docetaxel group compared to the NHT group (78.6% vs 25%, *p* value: 0.004).

### Biomarker analyses

**Fig. 2:**
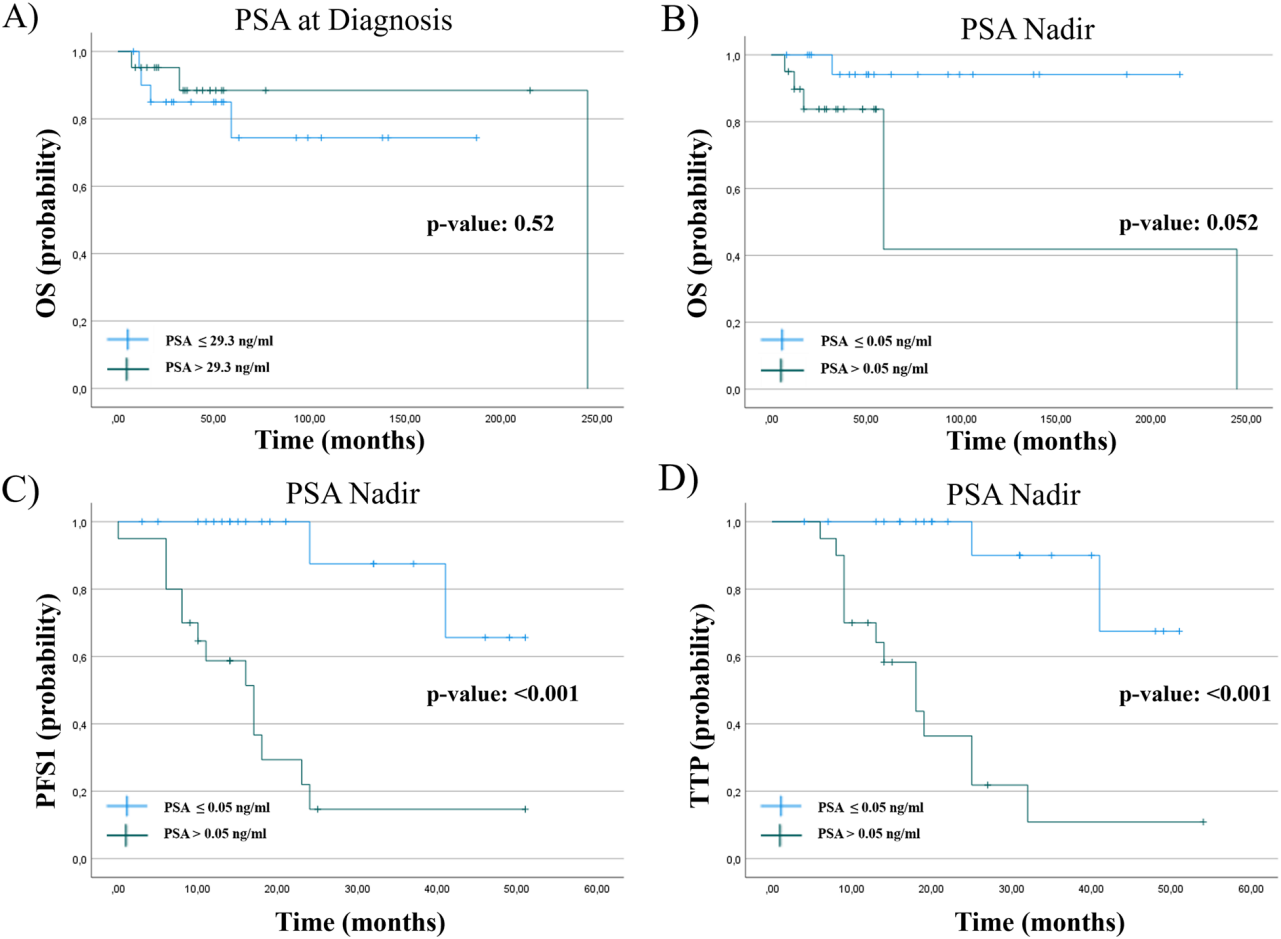
Kaplan-Maier curve of PSA at diagnosis and OS (*p* value: 0.52). PSA Nadir of ≤ 0.05 mg/ml related to **B** OS (*p* value: 0.52), **C** PFS1 (*p* value: < 0.001) and **D** TTP (*p* value: < 0.001)

Up to now, PSA still represents the most important serum biomarker for initial PCa detection and monitoring [7]. PSA Nadir, defined as the lowest measured PSA during therapy, represents an important marker for clinical response after local therapy as well as during systemic treatment [8, 9]. In our work the PSA-Nadir relates to the lowest PSA-value since beginning of the systemic therapy and not to the PSA after the initial local treatment. Importantly we found that the PSA level at primary diagnosis was not correlated to OS (*p* value: 0.52), PFS1 (*p* value: 0.55), or TTP (*p* value: 0.45). A PSA decline of more than 50% (PSA50) was achieved in the majority patients during therapy (91.7% NHT vs. 94.4% Docetaxel), implying that PSA50 often used in clinical trials is not a reasonable marker to predict treatment response in our population. Thus, we determined a PSA Nadir of ≤ 0.05 ng/ml as cut-off level and were able to demonstrate a significantly prolonged PFS1 and TTP (*p* value: < 0.001) in those patients who reached a PSA Nadir of ≤ 0.05 ng/ml during therapy (Fig. 2).

**Fig. 3:**
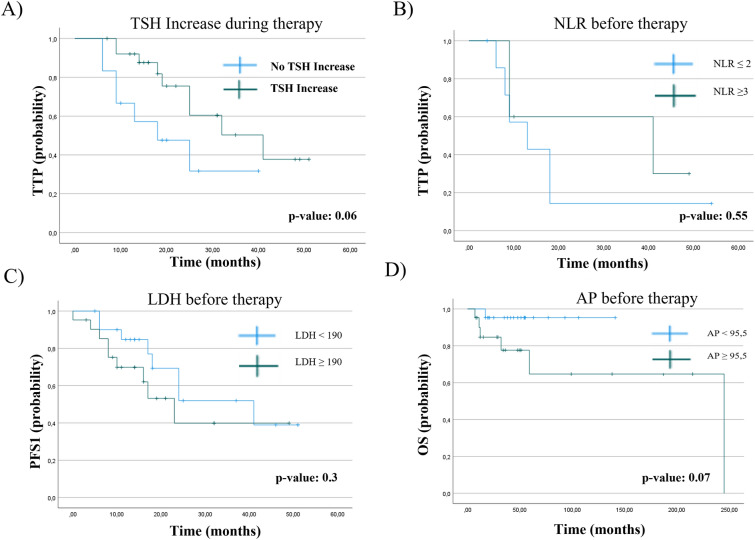
Kaplan-Maier curves of laboratory values before or during therapy and clinical outcome: **A** Relation of TSH increase and TTP (*p* value: 0.06). **B** Relation of Neutrophil lymphocyte ratio (NLR) and TTP (*p* value: 0.55). **C** Relation between elevated LDH (defined as > 190 U/L) and PFS1 (*p* value: 0.3). **D** OS correlated to level of AP (*p* value: 0.07)

Based on our previous study describing an increase in TSH levels as predictive biomarker for therapy response in metastatic castration resistant PCa (mCRPC) during AAP, we analyzed TSH changes in the present cohort [10]. TSH increase was defined as increase of any TSH levels within 3 months after initiation of treatment. A TSH increase was achieved in 76.2% of the NHT and in 58.8% of the Docetaxel group (*p* value: 0.2). Indeed, a TSH increase observed within 3 months after therapy start resulted in prolonged TTP (*p* value: 0.06, Fig. 3A). Next, we assessed the neutrophil–lymphocyte ratio (NLR) before and 4–6 weeks after treatment start as potential biomarker. However, in contrast to other studies [11, 12] we were not able to find a correlation of either pre-therapy NLR, or NLR change during therapy with OS (*p* value: 0.55), PFS (*p* value: 0.122) or TTP (*p* value: 0.113), Fig. 3B**)**. Furthermore, we could not demonstrate a correlation between lactate dehydrogenase levels (LDH) and OS (*p* value: 0.3), PFS1 (*p* value: 0.285) or TTP (*p* value: 0.51), Fig. 3C). Concerning alkaline phosphatase (AP) there was a trend towards a shorter OS for elevated AP levels before treatment start (*p* value: 0.07), Fig. 3D). Elevated LDH and AP levels before therapy were observed slightly more often in the Docetaxel group (LDH: 55.6% vs 45.8%, AP: 55.6% vs 45.8%). Analyzing various clinical parameters, we found that CCI was correlated to OS (*p*_cox-regression OS=_ 0.028) highlighting CCI as a prognostic factor also in mHSPC.

## Discussion

In the present small monocentric real-world study, we observed a clear trend towards a benefit of NHT + ADT compared to Docetaxel + ADT regarding OS, PFS and TTP in our study population. This finding is concomitant to a recent meta-analysis encompassing 7287 patients as well as summarized in a recent systematic review comprising the prospective phase III registration trials [13, 14]. However, there also exist data that treatment with NHT + ADT did not offer a statistically significant advantage in survival [15, 16]. Worth mentioning, most meta-analyses evaluate mainly data produced by registrations trials not reflecting the real-world situation as many patients represent with advanced age, decreased performance status or multimorbidity who would be excluded from clinical trials, again highlighting the importance of real-word assessments. In addition, one has to consider drug-drug interactions which is not always respected in daily practice influencing drug bioavailability in a significant proportion of patients [17]. Particularly new oral anticoagulants, which are often prescribed for patients with cardio-vascular disease have major interaction with NHT like, e.g. Apalutamide and Apixaban.

Certainly, our patient collective did not imply patients treated by Enzalutamide, the third NHT approved. Since Enzalutamide has been EMA approved in March 2021, follow-up of these patients was too short to calculate survival. In addition, due to the relatively small sample size we did not perform subgroup analyses, e.g. assessing patients stratified according to disease volume, an important aspect in determining the optimal treatment or even patients´ age [18]. In addition, longer follow-up and consecutive treatments would be of utmost clinical interest.

Generally, there is need of reliable biomarkers for selecting initial treatment options between ADT intensification with Docetaxel or NHT. Based on recent studies like PEACE-1 (ADT + Docetaxel + Abi) and ARASENS (ADT + Docetaxel + Darolutamide), triple combination therapies show benefits in OS and PFS [19, 21] suggesting that triple therapies will most likely become the new SOC in the high-volume patients in the near future. Hence, regarding this rapid change in first lines therapies, reliable biomarkers are of special importance for monitoring response and detecting treatment resistance. In reference to upcoming triple therapies, PSA Nadir ≤ 0.05 ng/ml as described in this manuscript might be investigated as selection parameter in patients who do not achieve this cut-off level with a dual treatment to intensify the first-line therapy by initiating a triple therapy. Therefore, we investigated in the present study clinical biomarkers predicting therapy response.

While total PSA level at diagnosis was no indicator for clinical outcome, we turned our attention to PSA Nadir and demonstrated a strong correlation to OS, PFS1 and TTP. These data are in line with data from the LATITUDE trial, demonstrating that a PSA Nadir ≤ 0.1 ng/ml is significantly correlated with a prolonged radiographic PFS and OS [20]. Comparable results were lately presented at the ASCO 2022 by collaborators of the ARASENS trial [21] showing that the achievement of undetectable PSA after 36 weeks was associated with improved OS reducing the risk of death by 63% compared to those who had detectable PSA at the same time. Strengthening this data, we were able to show a statistically significant prolonged PFS1 and TTP with the cut of level for PSA Nadir at ≤ 0.05 ng/ml in our study. In reference to upcoming triple therapies, PSA Nadir ≤ 0.05 ng/ml as described in this manuscript might be used as selection parameter in patients who do not achieve this cut-off level with a dual treatment to intensify the first-line therapy by initiating a triple therapy.

In contrast to other studies, we could not identify a correlation of LDH [22], AP [23] and NLR [11] to OS, PFS or TTP. However, the development of hypothyroidism during treatment seems to correlate to a treatment response as already shown by our group in the mCRPC setting [10]. Of note, this was observed more often in the NHT group (76.2% vs 58.8%). As mentioned above, we could demonstrate that CCI was associated with OS. According to the literature, there are studies depicting CCI as important predictor for OS after RPE [24, 25], whereas another retrospective analysis of 221 patient with mCRPC could not show a predictive value [26].

Summarizing we elaborated a PSA Nadir ≤ 0.05 ng/ml as well as an increase in TSH during therapy as prognostic biomarkers during combination therapy in mHSPC. Presently intensive research is ongoing to identify tissue- and blood-derived biomarkers to characterize clinical phenotypes in mHSPC (27) that will further help to strike a new path towards precision medicine also in metastatic PCa.

Although we postulated a strong hint toward a better outcome in the NHT group compared to the Docetaxel group, there are limitations of our work to be aware of. First, our results did not reach a statistical significance and so the findings do not allow to draw definitive conclusions without further research. While the baseline characteristics of the groups were statistically well balanced (apart from PSA-level at start of therapy) the analysis might have been underpowered to detect meaningful differences due to the moderate sample size. The small study population is presumably the major limitation of this work. In addition the not-randomized design of the study might lead to systematic differences within the groups which would confound the survival-endpoints. By performing a multivariable analysis adjusting for age and PSA at therapy start, we tried to diminish this problem. Interestingly the results of uni- and multivariate calculation did not differ.

In general our findings have to be evaluated in multicenter retrospective studies or even better in prospective, randomized trials.

## Conclusion

Our real-world analysis strengthens computational findings towards a benefit of a NHT (AAP/Apa) as first-line treatment compared to Docetaxel. PSA Nadir ≤ 0.05 ng/ml was a strong indicator for therapy response, moreover the development of hypothyroidism might be a clinical useful marker predicting therapy success.

## Abbreviations

AAP: Abiraterone acetate/prednisolone
ADT: Androgen deprivation therapy
AP: Alkaline phosphatase
Apa: Apalutamide
CCI: Charlson Comorbidity index
CSS: Cancer-specific survival
DRE: Digital rectal examination
EBRT: External beam radiation therapy
Enza: Enzalutamide
ISUP: International Society of Urological Pathology
LDH: Lactate dehydrogenase
mCRPC: Metastatic castration-resistant PCa
mHSPC: Metastatic hormone-sensitive PCa
NHT: Novel hormonal therapy
NLR: Neutrophil lymphocyte ratio
OS: Overall survival
PCa: Prostate cancer
PFS: Progression-free survival
PSA: Prostate specific antigen
RPE: Radical prostatectomy
HR: Hazard ratio
TTP: Time to progression
